# Clinical and prognostic features among children with acute encephalitis syndrome in Nepal; a retrospective study

**DOI:** 10.1186/1471-2334-11-294

**Published:** 2011-10-28

**Authors:** Ajit Rayamajhi, Imran Ansari, Elizabeth Ledger, Krishna P Bista, Daniel E Impoinvil, Sam Nightingale, Rajendra Kumar BC, Chandeshwor Mahaseth, Tom Solomon, Michael J Griffiths

**Affiliations:** 1 Department of Paediatrics, Kanti Children's Hospital, Maharajgunj, Kathmandu, Nepal; 2 Department of Pediatrics, National Academy of Medical Sciences, Kathmandu, Nepal; 3 Brain Infections Group, Department of Clinical Infection, Microbiology and Immunology, Institute of Infection and Global Health, University of Liverpool, UK; 4 Department of Paediatrics, Patan Academy of Health Sciences, Patan Hospital, Lalitpur, Kathmandu, Nepal; 5 LUCINDA Group, Department of Epidemiology and Population Studies, Institute of Infection and Global Health University of Liverpool, UK; 6 Nepal Health Research Council, Ram Shah Path, Kathmandu, Nepal

## Abstract

**Background:**

Acute encephalitis syndrome (AES) is commonly seen among hospitalized Nepali children. Japanese Encephalitis (JE) accounts for approximately one-quarter of cases. Although poor prognostic features for JE have been identified, and guide management, relatively little is reported on the remaining three-quarters of AES cases.

**Methods:**

Children with AES (n = 225) were identified through admission records from two hospitals in Kathmandu between 2006 and 2008. Patients without available lumbar puncture results (n = 40) or with bacterial or plasmodium infection (n = 40) were analysed separately. The remaining AES patients with suspected viral aetiology were classified, based on positive IgM antibody in serum or cerebral spinal fluid, as JE (n = 42) or AES of unknown viral aetiology (n = 103); this latter group was sub-classified into Non-JE (n = 44) or JE status unknown (n = 59). Bad outcome was defined as death or neurological sequelae at discharge.

**Results:**

AES patients of suspected viral aetiology more frequently had a bad outcome than those with bacterial or plasmodium infection (31% versus 13%; P = 0.039). JE patients more frequently had a bad outcome than those with AES of unknown viral aetiology (48% versus 24%; P = 0.01). Bad outcome was independently associated in both JE and suspected viral aetiology groups with a longer duration of fever pre-admission (P = 0.007; P = 0.002 respectively) and greater impairment of consciousness (P = 0.02; P < 0.001). A higher proportion of JE patients presented with a focal neurological deficit compared to patients of unknown viral aetiology (13/40 versus 11/103; P = 0.005). JE patients weighed less (P = 0.03) and exhibited a higher respiratory rate (P = 0.003) compared to Non-JE patients.

**Conclusions:**

Nepali children with AES of suspected viral aetiology or with JE frequently suffered a bad outcome. Despite no specific treatment, patients who experienced a shorter duration of fever before hospital admission more frequently recovered completely. Prompt referral may allow AES patients to receive potentially life-saving supportive management. Previous studies have indicated supportive management, such as fluid provision, is associated with better outcome in JE. The lower weight and higher respiratory rate among JE patients may reflect multiple clinical complications, including dehydration. The findings suggest a more systematic investigation of the influence of supportive management on outcome in AES is warranted.

## Background

Acute encephalitis syndrome (AES) is a constellation of clinical signs and/or symptoms, i.e. acute fever, with an acute change in mental status and/or new onset of seizures [1]. These clinical signs suggest the patient has acute inflammation of the brain and are used by clinicians to identify patients with acute encephalitis. Viruses are regarded as the most important cause of the acute encephalitis syndrome worldwide. However, the syndrome can be associated with a range of pathogens, including acute bacterial or parasitic infection. Where population based studies have been undertaken, the incidence ranges between 3.5 and 7.4 cases per 100,000 patient-years [2]. Acute encephalitis can be associated with severe complications, including impaired consciousness, seizures, limb paresis or death [3].

In Asia, the major identified cause of acute encephalitis is Japanese Encephalitis (JE) virus. JE affects over 50,000 people annually, leading to 8-30% mortality and 50-60% disability, with children bearing the brunt of the disease burden [1,4-6]. JE is associated with considerable mortality and morbidity among Nepali children [3]. Consequently, the Ministry of Health and Population of Nepal, supported by the office of Infection Prevention Division, World Health Organisation (WHO), has integrated JE surveillance with Acute Flaccid Paralysis, Neonatal Tetanus and Measles in its National surveillance network since 2004 [7]. Over 23,000 cases of AES and 2500 cases of JE have been reported by the WHO surveillance network since 2004 (personal communication: Mr Tika Sedai, Data Manager, Programme for Immunization Preventable Diseases, WHO, Kathmandu, Nepal).

In Nepal, like many countries throughout Asia, test results for JE are often not available until weeks after the patient presents to the health care centre, because they are performed in a centralized government facility. Consequently, health care workers attempt to distinguish JE from other causes of AES based on the patient's clinical features, so that they can focus attention on known complications, such as seizures, and avoid unnecessary treatments, such as antibiotics. However, this approach can be inaccurate, leading to sub-optimal or inappropriate management. There have been several publications relating admission clinical parameters to outcome among JE cases, and the identification of poor prognostic indicators has helped focus attention on treatable complications of infection [8-10]. However, relatively little work has been done identifying prognostic features among the Non-JE AES patients. We therefore decided to investigate for diagnostic and prognostic features that distinguish between JE and other causes of AES, in a retrospective review of all children with AES at two hospitals in Kathmandu, Nepal.

## Methods

The hospital records of all children, aged 1 - 14 years, presenting either to Kanti Children's Hospital, Maharajgunj, Kathmandu, Nepal or Patan Hospital, Lalitpur, Nepal, from January 2006 to January 2008 were screened for a history consistent with acute encephalitis syndrome (AES).

Kanti Children's Hospital is a busy tertiary level referral centre. It has 300 beds and provides health care services to 300-400 children per day. Patan Hospital is a general hospital situated at the southern end of Kathmandu. It has 450 beds and provides health services to around 1,000 people per day, predominantly adults.

The hospital notes were examined by qualified paediatricians employed within the respective hospitals. Relevant clinical features and laboratory parameters present at admission were recorded in a standardized proforma. Each proforma was designated a unique study number. Study data were transcribed from the proforma to the study database. The study number was used to access the data for all future data analyses.

The study was approved by the Instituitional Review Committee of Kanti Children's Hospital and Patan Hospital, Kathmandu, ethical committee of the Nepal Health Research Council, Kathmandu and the Ethical Review Committee of the Liverpool School of Tropical Medicine, Liverpool, UK.

### Acute encephalitis syndrome (AES) and Japanese encephalitis (JE) case definitions

The classification of AES was based on the World Health Organization's (WHO) definition [1]. The results of the JE testing, undertaken as part of the JE surveillance programme, were related to the identified cases. The AES cases, based on the results of their microbiological and serological tests, were classified as AES of suspected viral aetiology (Confirmed JE, Non-JE and JE Status unknown) and AES of non-viral aetiology (AES-bacterial or parasitic aetiology). The clinical features within each class were examined. Cases definitions were as follows:

• *AES*: Fever or recent history of fever with change in mental status (including confusion, disorientation, coma, or inability to talk) and/or new onset of seizures (excluding simple febrile seizures). Other early clinical findings could include an increase in irritability, somnolence or abnormal behaviour greater than that seen with usual febrile illness [1,3].

• *AES of suspected viral aetiology*: was defined by fulfilling the definition for AES (above) and having a discharge diagnosis of suspected viral encephalitis or menigo-encephalitis, supported by a CSF cell count < 1000 cells/mm^3 ^with a lymphocyte predominance and no positive identification of non-viral pathogens (e.g. bacteria or parasites) in the CSF or blood [3,11].

• *Confirmed JE: *A suspected case which is shown to have IgM antibodies (≥ 40 units) specific to JE virus in a single (CSF and/or serum) sample (or a rise in titres among paired samples) as detected by IgM-capture ELISA (see testing below).

• *Non-JE*: A suspected viral case which is shown to have an absence of IgM antibodies specific to JE virus based on a negative test for a single sample collected after the ninth day of illness or no change in titres in paired samples collected at least seven days apart.

• *JE Status unknown*: A suspected viral case which was either not tested for anti-JE IgM antibodies or had samples tested that were collected too early in illness course to confidently rule out JE (as defined above).

• *AES of unknown viral aetiology*: A suspected viral case which was not confirmed as JE; this group included both of the categories described above, i.e. Non-JE, and JE Status unknown.

• *AES of non-viral aetiology*: was defined by fulfilling the definition for AES (above) and either; (a) having a documented discharge diagnosis of suspected bacterial meningitis or meningo-encephaltis, supported by a CSF cell count > 1000 cells/mm^3 ^or a pleocytosis with a polymorph predominance and a raised CSF protein (> 0.45 g/L) [3,11]; or (b) having a positive identification of non-viral pathogen in CSF or blood. A positive identification of a non-viral pathogen was fulfilled by a positive Gram stain or bacterial culture from CSF; a positive bacterial culture from blood; a positive Widal test for *Samonella typhi *[1,4]; or observation of asexual *Plasmodium falciparum *parasites in peripheral blood smears [4,6].

### Other clinical definitions

Outcome at discharge was classified as good or bad. Good outcome was defined as being alive with no impairment of consciousness or neurological sequelae. Bad outcome was defined by death or neurological sequelae at discharge. Patients that self-discharged or were referred to another hospital prior to discharge were excluded from outcome analysis.

Neurological sequelae were defined by the presence of one or more of the following at discharge; impaired consciousness, weakness (monoparesis hemiparesis, quadriparesis), focal or generalized abnormal limb tone (hypertonia, hypotonia), focal or generalized abnormal limb reflexes (hypereflexia, hyporeflexia), diagnosis of new onset or recurrent seizures or new or recurrent extra pyramidal movement disorders [5].

### JE diagnostic test

JE virus exposure was tested by MAC-ELISA (IgM antibody capture-Enzyme Linked Immunosorbent Assay). The ELISA plates were supplied by the Armed Forces Research Institute of Medical Sciences (AFRIMS), Bangkok, Thailand [12]. ELISA measurements were undertaken following the protocol supplied by AFRIMS. Diluted patient sera (1:100) or CSF (1:10) were added to the plates. Absorbance of experimental, positive and negative control samples were measured in duplicate in 96-well plates using a micro-titre plate reader (HumaReader Single Plus, Human GmbH, Wiesbaden, Germany). Single experimental patient samples with a mean absorbance ≥ 40 units (following subtraction of the absorbance for the negative controls) were considered positive. External quality assurance was undertaken by AFRIMS [12].

### Statistical methods

The acquired data for all AES patients was initially validated, coded and entered in SPSS Statistics software version 17.0 (IBM-SPSS, New York) for analysis. Differences between clinical groups were compared using Student independent samples t-tests (for Normally distributed data), Mann-Whitney U-tests (for non-Normally distributed data) and Fisher Exact tests (for categorical data/proportions). All clinical feature variables (with the exception of all duration and numbers of episodes variables apart from duration of fever) were entered into a forward stepwise multiple logistic regression model to identify variables independently associated with bad outcome or JE positive status; multiple imputation methods (10 iterations) were used to overcome problems of missing observations and collinearity statistics were examined to ensure independence of the predictor variables. The median age and numbers of AES cases were mapped using ArcGIS version 9.3 (Esri Ltd., California) software to show their distribution in the different districts across Nepal in order to identify any spatial patterns in infection dynamics. Statistical significance was set at the conventional 5% level for all analyses.

## Results

### Baseline characteristics

A total of 225 children with acute encephalitis syndrome (AES) were admitted to the two hospitals between January 1^st^, 2006 and January 1^st^, 2008. Forty (18%) of these patients did not have any lumbar puncture (LP) results available in their notes. These patients were analysed separately.

**Figure 1:**
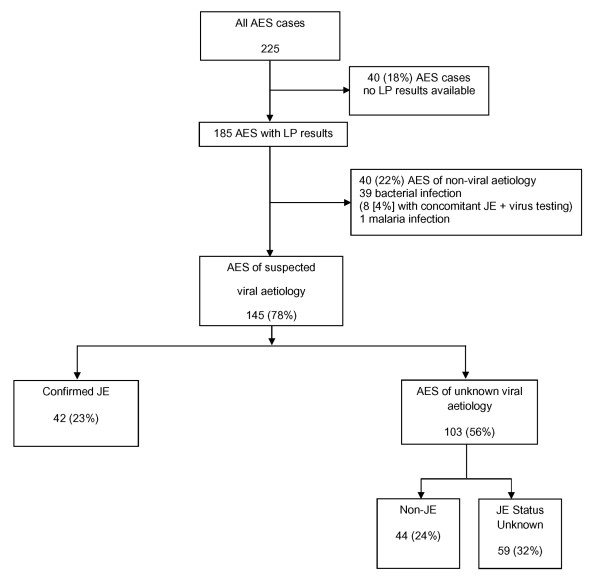
**Flow diagram of Acute Encephalitis Syndrome patients based on aetiological classification**. 225 children with Acute Encephalitis Syndrome (AES) were identified. To analyse AES of different aetiologies only patients where LP results were available were taken forward (n = 185). Patients with non-viral aetiologies were analysed separately (n = 40). The remaining AES patients were classified as JE (n = 42) or AES of unknown viral aetiology (n = 103) based on presence or absence of high anti-JE virus immunoglobulin titres. AES of unknown viral aetiology was further sub-classified into Non-JE (n = 44) and JE Status Unknown (n = 59) based on presence or absence of low or negative anti-JE virus immunoglobulin titres.

Of the remaining 185 AES patients, 40 (22%) were diagnosed with either bacterial (n = 39) or *Plasmodium falciparum *infection (n = 1). Eight of these patients also had elevated anti-JE virus titres (≥ 40 units) on serum IgM testing during acute illness. Since bacterial co-infection can change clinical features and influence patient outcome, these patients were analysed separately (Figure 1).

One hundred and forty five AES patients (145/185;78%) were considered to have AES of suspected viral aetiology. Forty-two of these patients (23%) were confirmed as JE. Among the other 103 patients, 44 tested negative to JE using samples collected after the ninth day of their illness and therefore were classified as Non-JE. For the remaining 59 patients JE status was unknown (Figure 1).

The majority of JE positive patients, 41/50 (82%), were identified by serum testing positive for anti-JEV IgM antibodies, with 31/41 (79%) identified by a single serum sample. The other serum samples tested positive in paired samples. The remaining 9 patients were diagnosed by testing positive for anti-JEV IgM antibodies in the CSF.

The majority of the non-viral AES patients were diagnosed with suspected bacterial meningitis based on a raised cell count with a polymorph predominance and raised protein in the CSF. The commonest identified cause of infection was gram stain positive bacteria in CSF.

**Figure 2:**
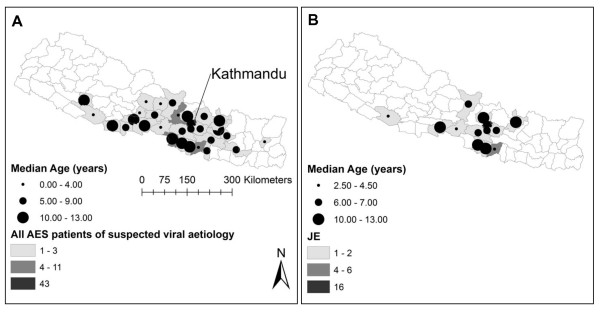
**Map of residence district for Acute Encephalitis Syndrome patients of suspected viral aetiology**. Panel A, All AES patients of suspected viral aetiology; Panel B, JE patients; Increasing depth of shading within a district indicates a higher number of AES patients were admitted from this district. Unshaded districts indicate no patients were admitted from this district. Increasing circle diameter within a district indicates AES patients of an older (median) age were admitted. Kathmandu is labelled on the map.

Patients presented to Kanti Children's (n = 208) or Patan hospital (n = 17) from a wide range of outlying districts from Kathmandu including the hill and mountain districts. There were no marked differences in geographic distribution for the number of cases or age at presentation among the different sub-groups of suspected viral AES patients (Figure 2). Route of presentation to hospital was documented in 175 AES patients. Self-referral was the commonest route of presentation, reported by 96/175 (55%) of AES patients.

### Patient outcome

Outcome at discharge was recorded for 183/185 patients where LP results were available and 38/40 patients without LP results. Among the patients without LP results, seventeen (45%) had a bad outcome; 10 (26%) died and a further 7 (18%) had neurological sequelae at discharge.

Among the AES patients with non-viral infection, 38/40 (95%) had an outcome at discharge recorded. Five (13%) had a bad outcome; 2 died and a further 3 had neurological sequelae at discharge. All 8 patients with bacterial and JE co-infection exhibited complete recovery at discharge. The one patient with P.falciparum infection recovered completely.

Among the AES patients with suspected viral infection, 45/145 (31%) had a bad outcome; 8 died and 37 had neurological sequelae at discharge. Among the sub-set of confirmed JE patients 20/42 (48%) had a bad outcome; 4 died and 16 had neurological sequelae (Table 1).

**Table 1:** Patient outcome for 6 aetiological categories of Acute Encephalitis Syndrome.

	Suspected viral aetiology															Non-viral aetiology		
	
Outcome	AES of suspected viral aetiology			Confirmed JE			AES of unknown viral aetiology			Non-JE			JE Status Unknown			Bacterial (n = 37)* P.f. (n = 1)		
Lp status	**+Lp**	-Lp	Both	**+Lp**	-Lp	Both	**+Lp**	-Lp	Both	**+Lp**	-Lp	Both	**+Lp**	-Lp	Both	**+Lp**	-Lp	Both
Patient No.	**145**	35	180	**42**	8	50	**103**	27	130	**44**	12	56	**59**	15	74	**38**	3	41
Good	100 (69)	19 (54)	119 (63)	22 (52)	3 (37.5)	25 (50)	78 (76)	16 (60)	94 (72)	22 (73)	8 (66)	40 (71)	46 (78)	8 (53)	54 (73)	33 (87)	2( 66)	35 (85)
Bad	45 (31)^§^	16 (46)	61 (37)^§^	20 (48)^‡¶^	5 (62.5)	25 (50)^‡¶∆^	25 (24)^¶^	11 (40)	36 (28)^¶^	12 (27)	4 (33)	16 (29)^¶∆^	13 (22)	7 (47)	20 (27)	5 (13)^§‡^	1 (33)	6 (15)^§‡^
Neurol. Sequelae	37 (25)	7 (20)	44 (24)	16 (38)	3 (37.5)	19 (38)	21 (20)	4 (15)	25 (19)	12 (27)	1 (8)	13 (23)	9 (15)	3 (20)	12 (16)	3 (8)	0 (0)	3 (7)
Died	8 (5)	9 (26)	17 (9)	4 (9.5)	2 (25)	6 (12)	4 (4)	7 (26)	11 (8)	0 (0)	3 (25)	3 (6)	4 (7)	4 (27)	8 (11)	2 (5)	1 (33)	3 (7)

Outcome at hospital discharge is presented for 2 main aetiological categories of **Acute Encephalitis Syndrome (**AES); suspected viral and non-viral. Based on JE serology, AES of suspected viral aetiology was split into Confirmed JE and Unknown viral aetiology. The latter group was sub-categorised into Non-JE and JE status unknown. AES of non-viral aetiology included Bacterial and Plasmodium falciparum (P.f.) infection. Within each category, patients were split into 3 groups based on availability of LP results; +Lp, those with LP results; -Lp, those without; Both, combining both +Lp and -Lp patients. Patients without LP results were assigned to an AES group based on their discharge diagnosis and JE serological results. Patients with a bad outcome were sub-classified into those with neurological sequelae at discharge or those that died. Number with (%) is presented. Significance was tested via Fisher's Exact test.

* 38/40 patients with AES of Non-viral aetiology had an outcome recorded at discharge.

^§ ^Significant difference between suspected viral and non-viral patients (P = 0.039 and P = 0.022 respectively for +Lp and Both groups).

^‡^Significant difference between JE and non-viral patients (P = 0.001 and P < 0.001 respectively for +Lp and Both groups).

^¶^Significant difference between JE and Unknown viral patients (P = 0.01 and P = 0.008 respectively for +Lp and Both).

^∆^Significant difference between JE and Non-JE patients in the Both group (P = 0.029).

A similar trend was observed for outcome between JE and Non-JE patients in the +Lp group (P = 0.074).

A higher frequency of death was consistently observed among -Lp patients compared to +Lp patients in each AES category.

A significantly higher proportion of patients without LP results died compared to those where LP results were available (10/38 [26%] versus 10/183 [5%]; P < 0.001). Similarly, the proportion of patients with bad outcome were higher among patients without LP results (17/38 [45%] versus 51/183 [28%]; P = 0.053). Exclusion of the patients without LP results may have influenced outcome within the AES groups. To help address this issue, an additional analysis was undertaken, whereby patients without LP results were classified into AES categories based on their hospital discharge diagnosis supported by their JE serological results. The patients contributed to all AES groups. Each AES category exhibited a higher rate of bad outcome. There were no significant changes in the proportion of patients with bad outcome between groups (Table 1).

A significantly higher proportion of patients with AES of suspected viral aetiology had a bad outcome compared to AES patients with a non-viral infection (45/145 [31%] versus 5/38 [13%]; P = 0.039). A significantly higher proportion of JE patients exhibited a bad outcome compared to AES patients of unknown viral aetiology (20/42 [48%] versus 25/103 [24%]; P = 0.01). A similar trend was observed when JE patients were compared to Non-JE patients, with a higher proportion of JE patients exhibiting a bad outcome (20/42 [48%] versus 12/44 [27%]); P = 0.07; Table 1).

### Prognostic features associated with bad outcome at discharge

Multiple parameters were associated with bad outcome at discharge for both AES cases of suspected viral aetiology (n = 145) and confirmed JE (n = 42) (Table 2). In both groups, bad outcome was associated with a longer duration of fever prior to admission, a lower Glasgow coma score, a focal neurological deficit, older patient age and higher weight at admission.

**Table 2:** Clinical features for JE and AES patients of suspected viral aetiology by outcome

	Confirmed JE (n = 42)			AES suspected viral aetiology (n = 145)		
	
Clinical Features at admission	Bad	Good	p-value	Bad	Good	p-value
No. patients	20	22		45	100	
Age (years)	10 (1-14) [20]	4.5 (1-13) [22]	0.004	8 (1-14) [45]	6 (1-14) [97]	0.005
Number of males	5 (25) [20]	11 (50) [22]	0.121	13 (29) [45]	30 (31) [98]	1.000
Fever prior to admission (days)*	7 (3-13) [20]	5 (1-12) [22]	0.010	7 (1-14) [45]	5 (1-13) [100]	< 0.001
Altered sensorium	17 (85) [20]	13 (59) [22]	0.091	39 (89) [44]	55 (56) [98]	< 0.001
Duration of altered sensorium (days)	2 (1-5) [13]	2 (1-3) [10]	0.839	2 (1-7) [30]	1 (1-4) [43]	0.009
Modified Glasgow coma score*	11 (5-15) [20]	14 (7-15) [22]	0.001	11 (3-15) [44]	14 (4-15) [98]	< 0.001
Focal neurological deficit	9 (50) [18]	4 (18) [22]	0.046	17 (40) [43]	7 (7) [100]	< 0.001
Any seizure prior to admission	10 (77) [13]	15 (75) [20]	1.000	24 (77) [31]	67 (83) [81]	0.591
Episodes of seizure prior to admission^‡^	2 (1-12) [9]	2 (1-4) [14]	0.723	2 (1-12) [19]	2 (1-7) [48]	0.034
Neck stiffness	14 (82) [17]	9 (45) [20]	0.040	29 (71) [41]	49 (54) [91]	0.086
Vomiting	10 (71) [14]	13 (87) [15]	0.390	29 (81) [36]	47 (86) [55]	0.573
Days of vomiting prior to admission^∆^	3 (2-10) [8]	3 (1-7) [9]	0.400	3.5 (1-12) [26]	2 (1-8) [37]	0.019
Weight (kgs)	19.5 (9-30) [20]	13 (1-26) [22]	0.017	19 (4-35) [45]	15 (1-70) [90]	0.040
Axillary temperature (°C)	38.1 (36.7-40.0) [20]	37.8 (36.7-39.4) [20]	0.170	37.8 (36.1-40.0) [44]	37.8 (36.1-40.0) [86]	0.256
Pulse rate (beats per min.)	101 (52-150) [20]	104 (76-130) [22]	0.905	106 (52-160) [44]	110 (64-170) [96]	0.972
Respiratory rate (breaths per min.)	28 (20-50) [20]	36 (20-80) [22]	0.064	28 (3-60) [45]	30 (16-90) [92]	0.158
Death prior to discharge	4 (20) [20]	0 (0) [22]	---	8 (18) [45]	0 (0) [100]	---
Neurological sequelae at discharge	16 (80) [20]	0 (0) [22]		37 (82) [45]	0 (0) [100]	
**Treatment**						
Phenytoin	6 (30) [20]	0 (0) [22]	0.007	18 (40) [45]	8 (8) [100]	< 0.001
Phenobarbitone	5 (25) [20]	3 (14) [22]	0.445	10 (22) [45]	10 (10) [100]	0.067
Dexamethasone	6 (30) [20]	4 (18) [22]	0.477	12 (27) [45]	22 (22) [100]	0.533
Mannitol	6 (30) [20]	2 (9) [22]	0.123	19 (42) [45]	15 (15) [100]	0.001

Median (range) or Number (%) [number of patients]. Significance of difference between groups by Fisher's Exact test or Mann-Whiney U test. ^‡^: patients with one or more seizures before admission only ^∆^: patients with vomiting prior to admission only * Parameters identified as independently associated with bad outcome in both AES groups.

To identify whether these features were independently associated with bad outcome, these variables were entered into a forward stepwise logistic regression model separately for each patient group. For the larger group, all AES of suspected viral aetiology, fever duration (P = 0.002), GCS (P < 0.001), and focal neurological deficit (P = 0.001) were retained in the model for outcome. In JE, fever duration (p = 0.007) and GCS (p = 0.020) were again independently associated with bad outcome (but not focal neurological deficit) together with age (P = 0.011).

**Figure 3:**
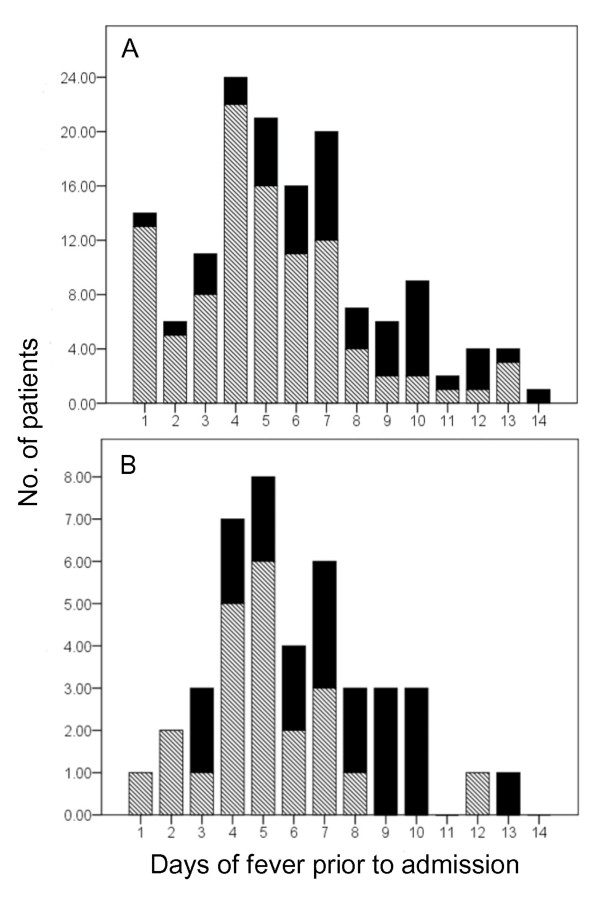
**Fever duration prior to admission organised by outcome among AES patients of suspected viral aetiology**. Panel A, All AES patients of suspected viral aetiology; Panel B, JE patients, X-axis; fever duration (1-14 days), Y-axis; Number of patients that presented to hospital at each day of fever duration (1-24), Solid bar shading; number of patients who exhibited a bad outcome at discharge, Hatched bar shading; number of patients who exhibited a good outcome at discharge.

To assess whether length of time from onset of illness to hospital admission was a prognostic marker for bad outcome, we used length of reported fever prior to admission as a proxy marker. We selected a threshold of 7 days of fever prior to admission (Figure 3). We found fever duration over 7 days was associated with a bad outcome. Fever duration was reported in 142/145 (98%) of patients with AES of suspected viral aetiology. Thirty-two patients reported fever duration of more than 7 days prior to admission. Of these, 19 had bad outcome and 13 had good outcome. The remaining 110 patients reported a fever duration under 7 days, of which 23 had bad outcome and 87 had good outcome (19/32 [59%] versus 23/110 [20%]; P < 0.001). This indicates that AES patients who present with a prolonged fever prior to hospital admission have an increased risk of having a bad outcome at discharge (Relative Risk = 2.8 [95% confidence interval: 1.6 - 4.4]).

Among the JE patients, a reported fever duration of more than 7 days prior to admission was again linked to a significantly higher rate of bad outcome compared to those who presented with a shorter duration of fever (9/11 [82%] versus 11/31[36%]; P = 0.013). Among patients with AES of non-viral aetiology, prolonged duration of fever prior to admission was also linked to a higher rate of bad outcome (2/6 [33%] versus 3/22 [14%]; P = 0.28).

Of the treatments given to AES patients of suspected viral aetiology, mannitol and phenytoin were more frequently prescribed among those with a bad outcome compared to those with a good outcome; 27/61 (44%) versus 19/119 (16%), P < 0.001 for mannitol; 23/61 (38%) versus 12/119 (10%), P < 0.001 for phenytoin, in bad and good outcome patients respectively.

### Clinical features that distinguish between confirmed JE and AES of suspected non-JE viral aetiology

Confirmed JE cases were compared to AES cases of unknown viral aetiology (Non-JE and JE status unknown). As the latter group also contained patients that did not have a confirmed negative test for JE, a further analysis was undertaken comparing JE against Non-JE cases.

The rates of many clinical features and laboratory parameters were similar on admission between confirmed JE, confirmed Non-JE and JE-status unknown (Tables 3 and 4). Importantly, the rates of many recorded neurological features were comparable between the patient groups. There were no significant differences in prevalence of an altered sensorium at admission, depth or duration of coma, frequency, duration or type of seizures between patient groups. However, there was a significantly higher prevalence of patients presenting with focal neurological deficits at admission among confirmed JE patients compared to AES cases of unknown viral aetiology (13/40 versus 11/103; P = 0.005; Relative Risk = 3.0 [95% confidence interval: 1.4-6.7]). Among AES patients of suspected viral aetiology, presence of a focal neurological deficit at admission had a positive predictive value of 32% (sensitivity 54%) for JE. Absence of a focal neurological deficit at admission had a negative predictive value of 89% (specificity 77%) for the patient not having JE.

**Table 3:** Clinical features at admission for 5 categories of Acute Encephalitis Syndrome

Clinical features	Confirmed JE	AES unknown viral aetiology (n = 103)		AES bacterial aetiology (n = 39)	
		Non-JE	JE Status Unknown	**Bact. Inf**.	Bact. and JEV+
No. patients (% AES patients (n = 184^†^))	42(23)	44(24)	59(32)	31(17)	8(4)
Age (years)	7(1-14)[42]	8(1-14)[44]	4.5(1-13)[56]	4.5(1-12)[31]	3.5(1-10)[8]
Number of males	16(38)[42]	16(36)[44]	16(27)[59]	11(35)[31]	8 [100][8]
Fever prior to admission (days)	5.5(1-13)[42]	6(2-14)[44]	5(1-12)[59]	5(1-13)[30]	5 (1-16)[7]
Altered sensorium	30(71)[42]	29(69)[42]	35(60)[58]	13(43)[31]	5 [63][8]
Duration of altered sensorium (days)	2(1-5)[23]	2(1-7)[24]	1(0-5)[27]	3(1-4)[9]	1(1-1.1)[3]
Modified Glasgow coma score	12(5-15)[42]	14(4-15)[44]	14(3-15)[59]	15(4-15)[30]	12(7-15)[8]
Focal neurological deficit	13(32)[40]	7(16)[44]	4(7)[59]	2(12.5)[16]	1(33)[3]
Any seizure prior to admission	25(76)[33]	25(78)[32]	42(89)[47]	21(75)[28]	4(50)[8]
Generalised seizure prior to admission	22(96)[23]	22(92)[24]	35(92)[38]	21(95)[22]	3(38)[8]
Focal seizure prior to admission	1(4)[23]	0(0)[24]	1(3)[38]	0(0)[22]	0(0)[8]
Episodes of seizure prior to admission	2(2-12)[23]	1(1-7)[17]	2(1-9)[27]	1.5(1-7)[18]	1.2 (1-1.5)[2]
Est. duration of longest seizure (mins.)	6(3-60)[16]	6(3-60)[16]	5(4-60)[12]	5(1-5)[3]	10(10-10.2)[2]
Neck stiffness	23(62)[37]	27(66)[41]	28(52)[54]	18(58)[31]	5(63)[8]
Vomiting	23(79)[29]	24(83)[29]	29(88)[33]	18(78)[23]	3(38)[8]
Days of vomiting prior to admission	3(1-10)[18]	4(1-11)[21]	2(1-12)[25]	3.5(1-10)[12]	1.2(1-2)[5]
Weight (Kg)	15.25(1-30)[42]	20(9-70)[38]*	14(1-60)[55]	15(1-60)[29]	11(1-25)[8]
Axillary temperature (°C)	37.8(36.7-40)[40]	37.2(36.1-40)[38]	37.8(36.1-40)[52]	37.8(36.7-41.1)[29]	38.9(37.8-39.4)[7]
Pulse rate (beats per min.)	101(52-150)[42]	100(69-160)[41]	110[78-170)[57]	100(70-160)[28]	120(72-130)[7]
Systolic BP (mmHg)	96(50-120)[17]	93(78-130)[20]	100(9-110)[14]	100(80-140)[11]	100(90-110)[3]
Dystolic BP (mmHg)	60(20-90)[17]	60(10-100)[20]	60(0-100)[15]	70(50-110)[11]	70(60-80)[3]
Respiratory rate (breaths per min.)	30(20-80)[42]	28(16-60)[40]**	30(3-90)[55]	34(20-50)[30]	34(24-40)[7]

Median (range) or Number (%) [number of patients]. Significance of difference between groups by Fisher's Exact test or Mann-Whiney U test. ^†^Single patient with *Plasmodium *infection not presented. Significant difference between JE and Non-JE patients; P = 0.031*; P = 0.003**

**Table 4:** Laboratory parameters at admission for 5 categories of Acute Encephalitis Syndrome

Laboratory parameters	Confirmed JE	AES unknown viral aetiology (n = 103)		AES bacterial aetiology (n = 39)	
		Non-JE	JE Status Unknown	**Bact**.	Bact. and JEV+
No. patients	42	44	59	31	8
**Blood**					
Hemoglobin (g/L)	112(80-190)[37]	116(80-140)[37]	112(60-200)[55]	116(30-460)[24]	112(81-120)[7]
Total leucocyte count (x10^9^/L)	10.9(2.46-33)[41]	9.6(1.8-25.8)[40]	12.5(4.0-9.8)[56]	9.5(1.48-110)[27]	12 (1.96- 190)[7]
Polymorphs (proportion)	0.73(0.08-0.92)[41]	0.72(0.3-0.96)[39]	0.75(0.16-0.93)[56]	0.77(0.50-0.92)[27]	0.82(0.63-0.95)[7]
Lymphocytes (proportion)	0.21(0.08-0.82)[41]	26(0.04-0.7)[39]	0.24(0.06-0.84)[54]	0.21(0.08-0.4)[27]	0.18(0.05-0.34)[7]
Blood sugar (mmol/L)	5.1(2.2-12.1)[13]	5.1(2.2-10.3)[20]	4.4(3.3-7.5)[26]	6.5(3.7-13.3)[4]	7.8(6.4-8.2)[5]
Urea (mmol/L)^†^	15(6.8-17.1)[5]	8.9(1.4-18.9)[10]	10.3(0.7-20.3)[10]	nr	9.6(5.3-21.4)[3]
Creatinine (μmol/L)	44.2(8.8-88)[3]	53.4(8.8-71.6)[11]	53.0(8.8-79.6)[13]	nr	64.5(35- 80)[3]
Sodium (mmol/L)	137(127-160)[12]	133(126-146)[17]	133(109-149)[20]	139(129-148)[9]	137 (134- 139)[2]
Potassium (mmol/L)	4.3(3-5)[12]	3.9(3-5)[17]	3.95(2-5)[20]	3.8(3.5-4.9)[9]	3.8 (3.6- 3.9)[2]
**Cerebrospinal fluid**					
Total leucocyte count (x10^9^/L)	0.043(0-0.6)[42]	0.041(0-0.83)[44]	0.035(0-0.54)[59]	0.14(0-1.4)[31]*	0.07 (0-0.4)[8]
Polymorphs (proportion)	0.1(0-1)[41]	0.3(0-1)[42]	0.26(0-1)[57]	0.7(0-1)[30]*	0.8(0-0.9)[8]
Lymphocytes (proportion)	0.5(0-1)[41]	0.38(0-1)[42]	0.6(0-1)[57]	0.3(0-1)[30]	0.15(0-1)[8]
Protein (g/L)	0.4(0-1.2)[39]	0.4(0-1.5)[40]	0.4(0.1-1.5)[53]	0.65(0.2-1.5)[30]*	0.1(0.04-5.8)[8]
Sugar (mmol/L)	3.3(1.4-5.7)[41]	3.3(0.9-5.6)[43]	3.3(1.7-6.1)[53]	2.1(1.1-3.8)[30]*	2.1(0.7- 3.3)[8]

Median (range) or Number (%) [number of patients]. Significance of difference between groups by Fisher's Exact test or Mann-Whiney U test. *Significant (P < 0.01) difference between patients within AES of suspected viral aetiology and AES of bacterial aetiology; nr, not recorded; ^†^normal range for urea (newborn to 16 years): 1.1-6.4 mmol/L [13].

JE patients presented with a lower body weight (15.25 versus 20 Kg; P = 0.031), and a higher respiratory rate (30 versus 28 breaths per minute; P = 0.003) compared to confirmed non-JE patients. With the caveat that serum urea, creatinine and electrolytes were measured in only 25 AES patients of suspected viral aetiology, there was a trend for higher serum urea (15 versus 8.9 mmol/L; P = 0.27), sodium (137 versus 133 mmol/L; P = 0.09) and potassium (4.3 versus 3.9 mmol/L; P = 0.21) among JE compared to Non-JE patients (Table 4[13]). These parameters again showed a non-significant trend for higher median values among JE patients compared to AES patients of unknown viral aetiology (Table 4).

Both weight and respiratory rate, which were significantly different among JE patients compared to Non-JE patients, are in part dependent on patient age. To further dissect the influence of these parameters, age, weight and respiratory rate were entered into a stepwise logistic regression model. Only raised respiratory rate (P = 0.011) was retained.

**Figure 4:**
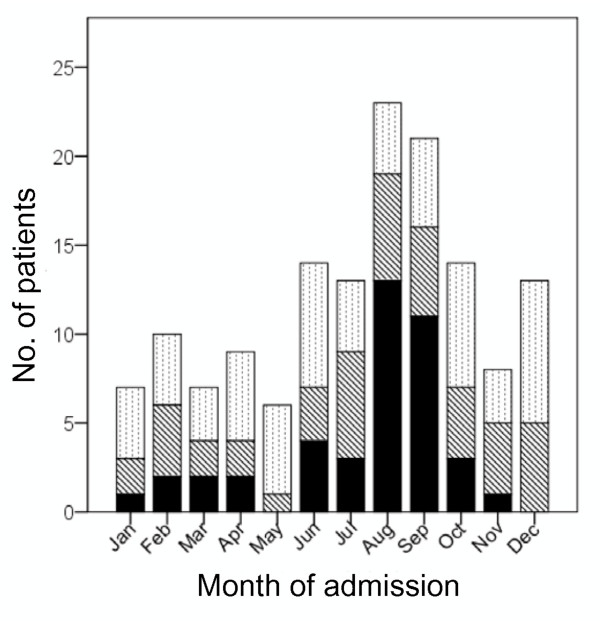
**Monthly admission numbers for Acute Encephalitis Syndrome (AES) patients of suspected viral aetiology**. X-axis; month of admission, Yaxis; Number of patients admitted to hospital each month, Solid bar shading; number of JE patients, Hatched bar shading; number of Non-JE patients, Dotted bar shading; number of patients where JE Status unknown.

Admission patterns also differed between JE and Non-JE patients. JE patients demonstrated a clear peak in hospital admissions rates in the months immediately following the rainy seasons each year (August and September in 2006-2007). Non-JE patients didn't demonstrate any clear seasonal variation in hospital admission rates (Figure 4).

## Discussion

This study demonstrates that patients with AES of suspected viral aetiology, either where JE was confirmed or where viral aetiology remained unknown, were significantly more likely to have a bad outcome compared to AES patients with bacterial or malaria infection.

Appropriately, JE surveillance is a health priority in Nepal. However, public health and clinical teams should be aware that patients with AES of unknown viral aetiology also have a high risk of morbidity and mortality. Furthermore, since there are up to 3 times more Nepali children with AES of unknown viral aetiology than proven JE, a bad outcome among the former group impacts on a larger number of children Therefore, identification and optimizing management of patients with AES should also be a priority.

The lower frequency of bad outcome among the AES patients with bacterial or malaria infection is likely to reflect the availability and effective use of antibiotics and anti-malaria treatment to reduce morbidity among these patients.

AES patients without LP results exhibited a significantly higher rate of death. The finding is likely to reflect lumbar punctures being undertaken less frequently on children who were critically ill. The finding highlights that restricting analyses to patients where LP results are available can lead to an underestimation of the frequency of death and bad outcome linked to AES.

Interestingly, the study has identified that the number of days of fever (reflecting number of days of illness) the patient experienced prior to hospital admission is a prognostic indicator of bad outcome in both patients with AES of unknown viral aetiology and JE. When analysis was applied to AES patients of bacterial aetiology no significant association was identified. Antibiotic use in the community may reduce fever duration prior to hospital admission in patients with bacterial infection and may confound the association between fever duration and bad outcome. In contrast, antibiotics would have limited impact on fever duration during viral infection.

Shorter duration of illness (or fever) prior to admission has previously been associated with good outcome among children for a range of diseases [8]. What is striking about our finding is that there is no specific treatment for JE or AES of suspected viral aetiology, yet attending hospital earlier in the illness course appears to be of benefit. The findings would suggest that hospital admission and the supportive management received there improves outcome.

Patients with impaired consciousness are often unable to drink themselves. Consequently, dehydration and metabolic acidosis may complicate AES [14]. Dehydration and acidosis would fit the significantly lower body weight and higher respiratory rate observed among JE compared to Non-JE patients. A previous prospective survey of JE management identified that fluid supplementation was associated with a positive influence on outcome [15]. Although appropriate fluid provision is a delicate balance when managing brain injury [16,17], one possible explanation of the positive influence of hospital admission may be that patients receive fluid support in hospital during the illness.

Given the commonest reported mode of presentation was self-referral, the focus on hastening patient attendance would lie with improving patient awareness of the features of AES and encouraging families to attend hospital promptly. Further research is needed to understand the factors that underlie families' health-seeking behaviours when a child is ill.

As shown previously, a low Glasgow coma scale (GCS) and/or a focal neurological deficit at hospital admission were independent clinical markers of bad outcome among children with AES [18]. JE patients frequently exhibit raised intra-cranial pressure, brain herniation syndromes [5] and focal brain lesions on neuro-imaging studies [19], explaining the preponderance of focal neurolgical deficits in this group.

In line with previous reports, mannitol was prescribed significantly more frequently among AES patients who exhibited a bad outcome [15]. Interestingly, a randomized clinical trial of mannitol among children with raised raised ICP secondary to cerebral malaria (another cause of non-traumatic brain injury) did not identify any beneficial effect [20]. A similar study would help clarify whether mannitol is a useful supportive treatment in AES [21].

JE patients exhibited an increased respiratory rate compared to Non-JE patients. Furthermore, JE patients who exhibited a high respiratory rate were associated with a good outcome, while those with a lower respiratory rate were associated with a bad outcome. This pattern can be observed during evolution of many complications, including metabolic acidosis, pneumonia, acute flaccid paralysis (involving the inter-costal muscles) or brain damage, where there may be an intial compensatory rise in respiratory rate followed by a fall when the body decompensates.

Tachypnea is also a feature of the severe brain injury syndrome - paroxysmal autonomic instability with dystonia (PAID)[22]. Patients with this brain injury syndrome exhibit intermittent agitation, diaphoresis, hyperthermia, hypertension, tachycardia, tachypnea, and extensor posturing. All of these signs overlap with features reported in both JE and AES. PAID may exist among AES patients.

As a descriptive analysis this study cannot distinguish cause from consequence. Consequently although several clinical features and interventions appear linked with outcome, this study is unable to determine whether these parameters are causal. Similarly, as a retrospective study it is limited by the breadth and quality of information available in the hospital notes. More detailed information on acid-base status would help discriminate between respiratory and metabolic causes of tachypnea. Similarly, more systematic measurement of urea and electrolytes and additional indicators of fluid balance would help assess the influence of fluid support on outcome. Based on their discharge diagnosis, the AES patients without LP contributed to all AES groups. However, a prospective study with a more systematic investigation of pathogen aetiology is warranted to confirm these findings.

## Conclusions

Nepali children with AES of suspected viral aetiology, either where JE is confirmed or where the aetiology remains unknown, exhibit a high rate of death and morbidity.

One of the more striking findings from the study was the association between long duration of fever prior to admission and bad outcome. If patients with AES of suspected viral aetiology, including those with confirmed JE, attend hospital early they are more likely to make a full recovery. Despite no specific treatment for JE or for patients with AES of suspected viral aetiology, the current management in Nepal can limit the development of neurological sequelae. The findings imply that family members, primary and community health care workers should be aware of AES and seek early referral for appropriate and potentially life-saving, supportive management.

Despite many comparable neurological features between JE patients and patients with AES of unknown viral aetiology, significantly more JE patients exhibited a bad outcome. This, in part, may reflect the higher proportion of JE patients that presented with a focal neurological deficit at hospital admission.

Further research is needed to understand the factors that underlie bad outcome in AES and JE, including a more systematic investigation of the influence of supportive measures.

## List of abbreviations

AES: Acute Encephalitis Syndrome; AFRIMS: Armed Forces Research Institute of Medical Sciences; CNS: Central Nervous System; CSF: cerebral spinal fluid; GCS: Glasgow Coma Scale; ICP: Intra-cranial pressure; JE: Japanese Encephalitis; LP: lumbar puncture; MAC-ELISA: IgM antibody capture-Enzyme Linked Immunosorbent Assay; PAID: paroxysmal autonomic instability with dystonia; WHO: World Health Organization.

## Competing interests

The authors declare that they have no competing interests.

## Authors' contributions

AR, TS and MJG conceived and designed the study. AR, IA, KPB, EL and SN acquired and undertook initial analysis of the data. AR, DI, RKBC, CM and MJG interpreted the data. AR, TS and MJG wrote the manuscript. All authors read and approved the final manuscript.

## Pre-publication history

The pre-publication history for this paper can be accessed here:

http://www.biomedcentral.com/1471-2334/11/294/prepub

## References

[B1] Solomon T Thao TT Lewthwaite P Ooi MH Kneen R Dung NM White N A cohort study to assess the new WHO Japanese encephalitis surveillance standards Bull World Health Organ 2008 86 3 178 186 10.2471/BLT.07.043307 18368204 PMC2647413

[B2] Granerod J Crowcroft NS The epidemiology of acute encephalitis Neuropsychol Rehabil 2007 17 4-5 406 428 10.1080/09602010600989620 17676528

[B3] Rayamajhi A Singh R Prasad R Khanal B Singhi S Study of Japanese encephalitis and other viral encephalitis in Nepali children Pediatr Int 2007 49 6 978 984 10.1111/j.1442-200X.2007.02495.x 18045307

[B4] Rayamajhi A Singh R Prasad R Khanal B Singhi S Clinico-laboratory profile and outcome of Japanese encephalitis in Nepali children Ann Trop Paediatr 2006 26 4 293 301 10.1179/146532806X152818 17132294

[B5] Solomon T Dung NM Kneen R Thao le TT Gainsborough M Nisalak A Day NP Kirkham FJ Vaughn DW Smith S Seizures and raised intracranial pressure in Vietnamese patients with Japanese encephalitis Brain 2002 125 Pt 5 1084 1093 11960897 10.1093/brain/awf116

[B6] Solomon T Dung NM Wills B Kneen R Gainsborough M Diet TV Thuy TT Loan HT Khanh VC Vaughn DW Interferon alfa-2a in Japanese encephalitis: a randomised double-blind placebo-controlled trial Lancet 2003 361 9360 821 826 10.1016/S0140-6736(03)12709-2 12642049

[B7] Pant GR A serological survey of pigs, horses, and ducks in Nepal for evidence of infection with Japanese encephalitis virus Ann N Y Acad Sci 2006 1081 124 129 10.1196/annals.1373.013 17135501

[B8] Solomon T Dung NM Kneen R Gainsborough M Vaughn DW Khanh VT Japanese encephalitis J Neurol Neurosurg Psychiatry 2000 68 4 405 415 10.1136/jnnp.68.4.405 10727474 PMC1736874

[B9] Libraty DH Nisalak A Endy TP Suntayakorn S Vaughn DW Innis BL Clinical and immunological risk factors for severe disease in Japanese encephalitis Trans R Soc Trop Med Hyg 2002 96 2 173 178 10.1016/S0035-9203(02)90294-4 12055808

[B10] Kumar R Mathur A Kumar A Sharma S Chakraborty S Chaturvedi UC Clinical features & prognostic indicators of Japanese encephalitis in children in Lucknow (India) Indian J Med Res 1990 91 321 327 2176644

[B11] Berkley JA Mwangi I Ngetsa CJ Mwarumba S Lowe BS Marsh K Newton CR Diagnosis of acute bacterial meningitis in children at a district hospital in sub-Saharan Africa Lancet 2001 357 9270 1753 1757 10.1016/S0140-6736(00)04897-2 11403812

[B12] Innis BL Nisalak A Nimmannitya S Kusalerdchariya S Chongswasdi V Suntayakorn S Puttisri P Hoke CH An enzyme-linked immunosorbent assay to characterize dengue infections where dengue and Japanese encephalitis co-circulate Am J Trop Med Hyg 1989 40 4 418 427 2540664 10.4269/ajtmh.1989.40.418

[B13] Behrman REKR Jenson HB Nelson Textbook of Paediatrics 2004 17 Philadelphia: Saunders

[B14] Solomon T Koelemay K Marfin A Roth C Jacobson J Ooi MH Rao N Sabchareon A Namghyal P Hills S Guidelines for management of children presenting with symptoms or signs of acute encephalitis syndrome Japanese Encephalitis Clinical Care Guidelines 2005 PATH

[B15] Tiroumourougane SV Raghava P Srinivasana S Badrinath Management parameters affecting the outcome of Japanese encephalitis J Trop Pediatr 2003 49 3 153 156 10.1093/tropej/49.3.153 12848204

[B16] Clifton GL Miller ER Choi SC Levin HS Fluid thresholds and outcome from severe brain injury Crit Care Med 2002 30 4 739 745 10.1097/00003246-200204000-00003 11940738

[B17] Gwer S Gatakaa H Mwai L Idro R Newton CR The role for osmotic agents in children with acute encephalopathies: a systematic review BMC Pediatr 2010 10 23 10.1186/1471-2431-10-23 20398408 PMC2859077

[B18] Klein SK Hom DL Anderson MR Latrizza AT Toltzis P Predictive factors of short-term neurologic outcome in children with encephalitis Pediatr Neurol 1994 11 4 308 312 10.1016/0887-8994(94)90007-8 7702691

[B19] Dung NM Turtle L Chong WK Mai NT Thao TT Thuy TT Kneen R Phu NH Wills B Farrar J An evaluation of the usefulness of neuroimaging for the diagnosis of Japanese encephalitis J Neurol 2009 10.1007/s00415-009-5249-5 19633907

[B20] Namutangula B Ndeezi G Byarugaba JS Tumwine JK Mannitol as adjunct therapy for childhood cerebral malaria in Uganda: a randomized clinical trial Malar J 2007 6 138 10.1186/1475-2875-6-138 17958887 PMC2147028

[B21] Kumar G Kalita J Misra UK Raised intracranial pressure in acute viral encephalitis Clin Neurol Neurosurg 2009 111 5 399 406 10.1016/j.clineuro.2009.03.004 19372001

[B22] Blackman JA Patrick PD Buck ML Rust RS Jr Paroxysmal autonomic instability with dystonia after brain injury Arch Neurol 2004 61 3 321 328 10.1001/archneur.61.3.321 15023807

